# Surgical management of a plexiform neurofibroma in neurofibromatosis type 1: The haemostatic challenge

**DOI:** 10.1016/j.jpra.2026.08.013

**Published:** 2026-08-19

**Authors:** Marta Matos, Katya Remy, Salim Zenkhri, Yves Harder, Julie Triolo

**Affiliations:** a Department of Plastic, Reconstructive and Aesthetic Surgery and Hand Surgery, Centre Hospitalier Universitaire Vaudois (CHUV), University of Lausanne (UNIL), Lausanne, Switzerland; b Faculty of Biology and Medicine, University of Lausanne (UNIL), Lausanne, Switzerland

**Keywords:** Neurofibromatosis type 1, Plexiform neurofibroma, Vascular anomalies, Hemostasis

## Abstract

**Background:**

Plexiform neurofibromas (PNFs) are benign tumours associated with neurofibromatosis type 1 (NF1) that may cause significant functional and cosmetic impairment. Surgical management is challenging because of diffuse infiltration and marked vascularity, increasing the risk of severe intraoperative bleeding.

**Case report:**

A 70-year-old woman with NF1 presented with a progressively enlarging mass of the left flank causing contour deformity with functional impairment. Previous resection 20 years earlier had been complicated by major haemorrhage requiring transfusion of red blood cells. MRI demonstrated a 40 × 25 cm subcutaneous infiltrative PNF without neither muscular nor neurological involvement. Following multidisciplinary discussion, partial surgical debulking was performed using a hemicircular dermolipectomy approach. Intraoperative haemostasis was complicated because of diffuse vascular abnormalities and extensive infiltrative growing pattern of the tumour. Visible bleeding was mainly controlled using vascular clips. Postoperatively, tranexamic acid and incisional negative pressure wound therapy were used. Symptomatic postoperative anaemia required transfusion of red blood cells. Recovery was otherwise uneventful, and histopathology confirmed benign neurofibroma without atypia. At 9 months, functional and cosmetic outcomes were satisfactory without recurrence.

**Conclusion:**

PNFs in NF1 patients remain surgically challenging because of their infiltrative growth and abnormal vascularity. Careful perioperative planning and meticulous haemostatic strategies are essential to reduce bleeding-related morbidity and optimize outcomes.

## Introduction

Neurofibromatosis type 1 (NF1) is an autosomal dominant disorder affecting approximately 1 in 3000 individuals.^1^ It is caused by mutations in the NF1 gene encoding neurofibromin, a tumour suppressor protein. Clinical manifestations include café-au-lait macules, axillary freckling, Lisch nodules, skeletal abnormalities, and neurofibromas.^1^^,^^2^

Plexiform neurofibromas (PNFs) occur in 30–50% of patients with NF1 and are considered pathognomonic for the disease.^2^ These tumours originate from peripheral nerves and grow in a diffuse and infiltrative pattern, involving adjacent soft tissues. PNFs may cause severe disfigurement, pain, and eventually functional impairment. These lesions also possess the potential for malignant transformation into malignant peripheral nerve sheath tumours.^3^

Surgical resection remains the cornerstone treatment for symptomatic PNFs. However, complete resection is often impossible because of extensive infiltration and proximity to critical structures. Furthermore, PNFs are associated with abnormal, fragile vasculature, predisposing patients to severe intraoperative bleeding.^4^, ^5^, ^6^, ^7^ Achieving adequate haemostasis, therefore, represents a major operative challenge.

We present the case of an elderly patient with a recurrent giant PNF of the flank, focusing on perioperative haemostatic management.

## Case report

**Fig. 1:**
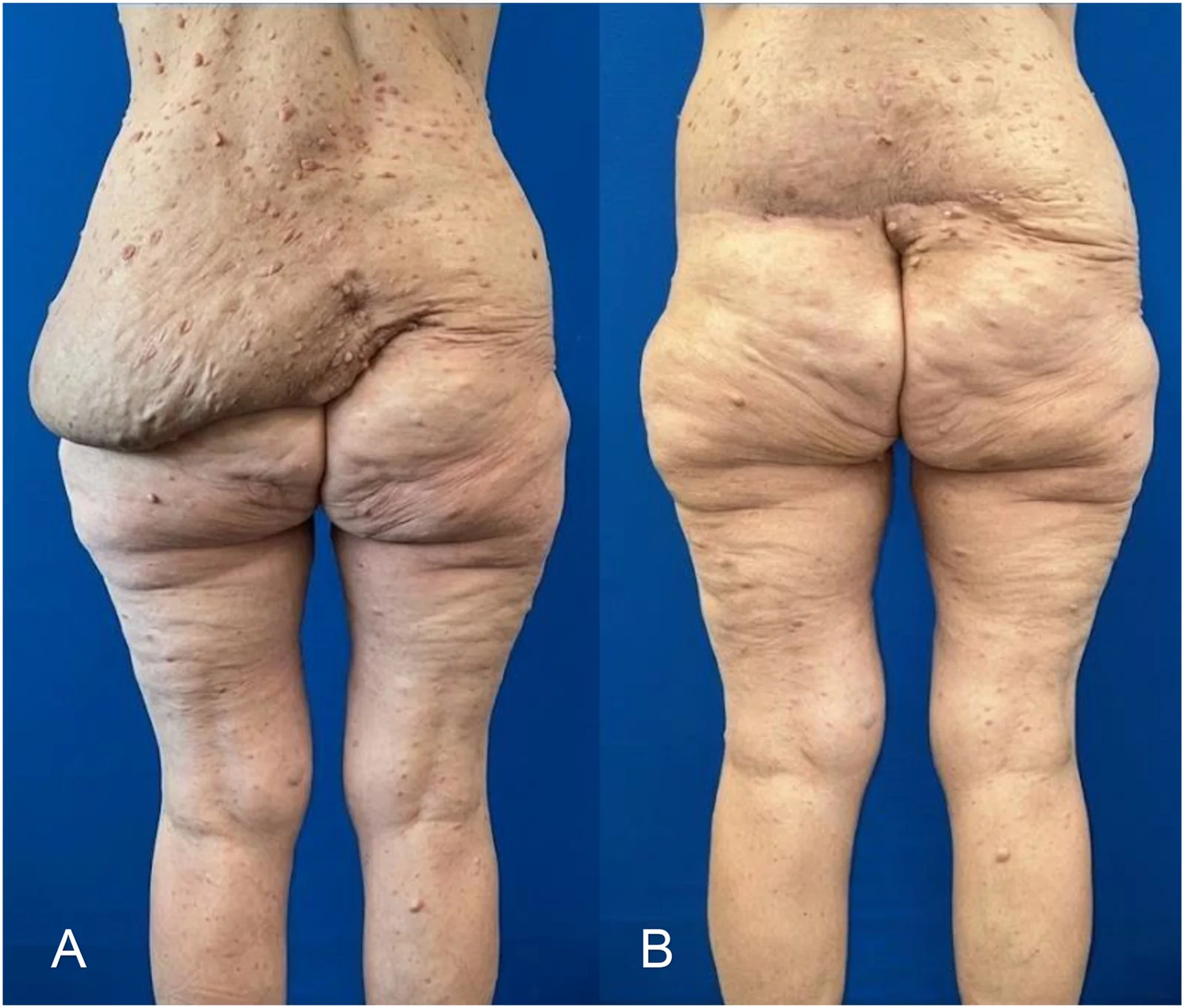
**Patient view, pre- and post-surgery:** Preoperative dorsal view of the patient with a large plexiform sagging mass compatible with extended neurofibroma located at the left flank causing significant contour deformity (A). Postoperative view at 3 months following surgery with restored contour and hardly visible scar (B).

A 70-year-old woman with known NF1 was referred for evaluation of a progressively enlarging isolated and recurrent soft-tissue mass of the left flank evolving over three years (Fig. 1a). The lesion caused major contour deformity and intermittent discomfort without neurological symptoms or constitutional signs. Twenty years earlier, resection of the same lesion at another institution had been complicated by severe intraoperative haemorrhage requiring blood transfusion.

She was known for hypercholesterolemia treated with statins. She had no coagulation disorders or smoking history. Clinical examination demonstrated a large mobile fibrous subcutaneous mass measuring approximately 40 × 20 cm. The overlying skin was intact without ulceration or signs of inflammation.

**Fig. 2:**
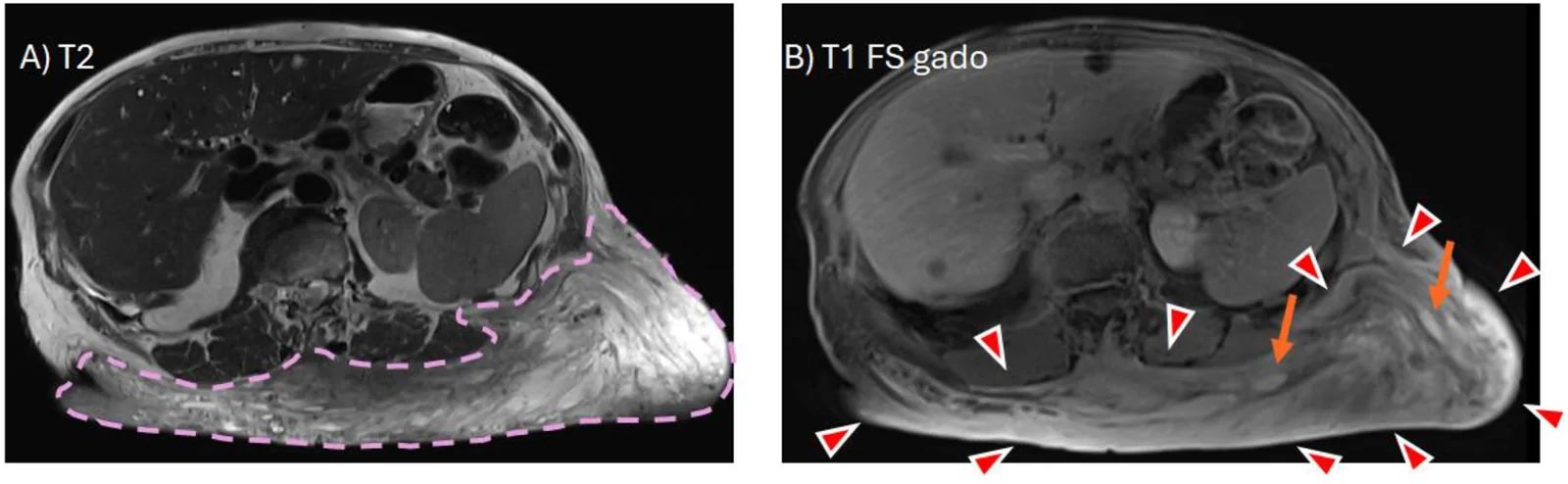
**T2-weighed axial MRI view** demonstrating extended, diffuse and infiltrative growing of a subcutaneous mass compatible with neurofibroma involving dorsum and left flank with contralateral extension to the right dorsal region (interrupted pink line) (A). **T1-weighted axial MRI view with intravenous gadolinium contrast media** showing heterogeneous subcutaneous contrast enhancement of the soft tissue mass (red arrowheads), without evidence of intra-abdominal extension. Presence of enhancing nodules within the soft-tissue mass, compatible with neurofibromatous foci (orange arrows) (B).

Laboratory investigations were normal with haemoglobin of 127 g/L. MRI confirmed a large infiltrative subcutaneous mass extending dorsally toward the left flank, consistent with plexiform neurofibroma (Fig. 2a). Radiologically, there was no muscle infiltration, spinal compression, or suspicious malignant component. Following multidisciplinary discussion involving plastic surgery, radiology, and sarcoma oncology specialists, surgery was indicated without a preoperative soft-tissue biopsy.

The patient underwent sub-total resection under general anaesthesia in a right lateral decubitus position using a left hemicircular dermolipectomy approach. The objective was debulking of the most deforming component while preserving deeper uninvolved structures. Tumour infiltration remained limited to subcutaneous tissue without extension beyond the fascia of the underlying muscles.

**Fig. 3:**
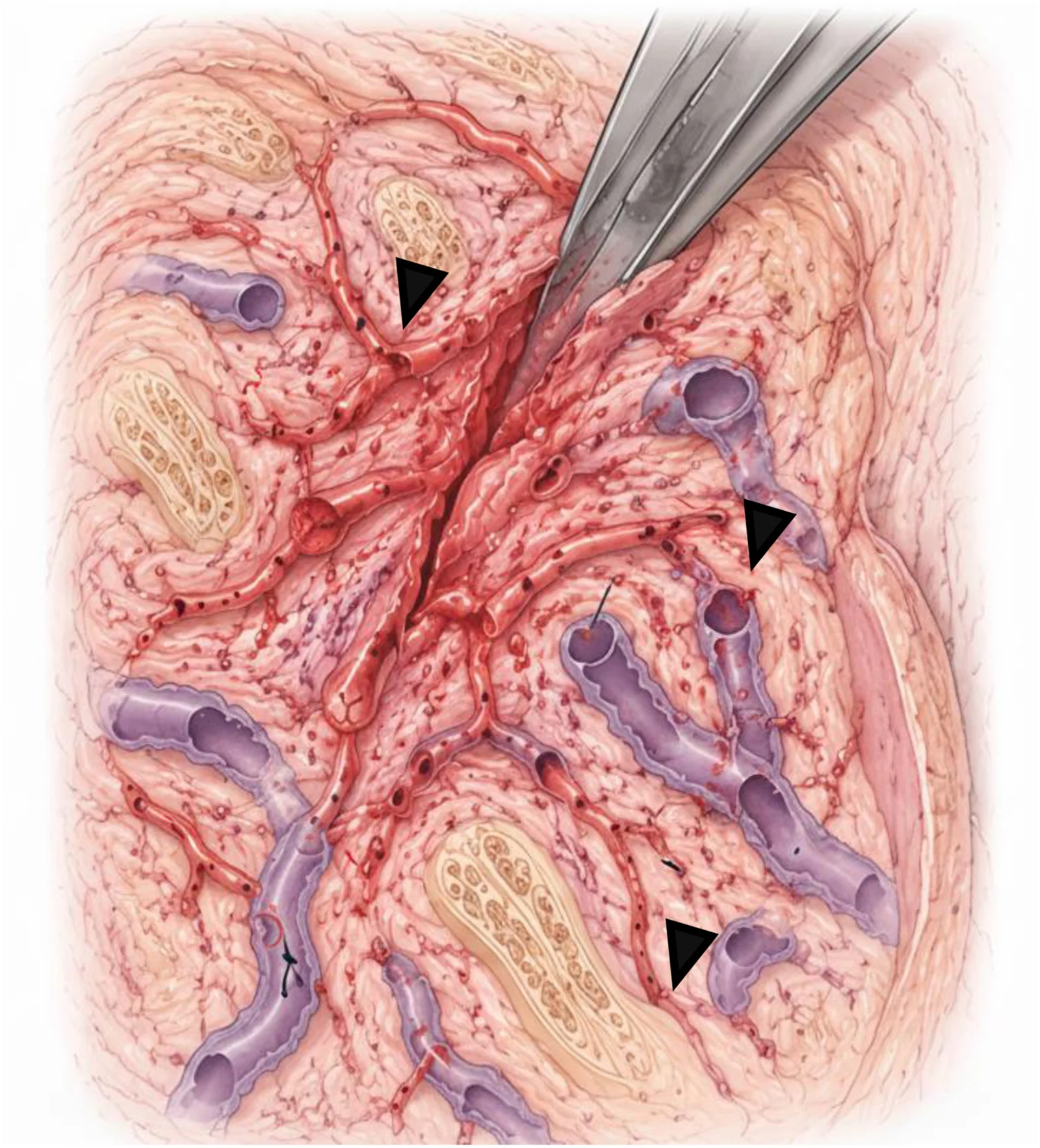
**Intraoperative anatomical illustration demonstrating the vascular architecture of PNF.** Numerous arterial and venous channels are diffusely distributed throughout the tumour and intimately associated with adjacent nerve fascicles. Fragile thin-walled vascular structures are prone to tearing during dissection, resulting in extensive bleeding points and difficult haemostasis. Arrows highlight representative intra-tumoral arteries and veins and their close relationship with neural elements.

The lesion was poorly demarcated and highly vascularized, containing glomeruloid clusters of tortuous and dysplastic vessels (Fig. 3). Haemostasis proved technically demanding because surgical vessel isolation was not possible due to infiltrative growing pattern of the soft tissue adjacent to the vessel’s walls. Electrocautery was primarily used for dissection and bleeding control of small to medium superficial vessels. However, numerous medium- to large-sized vessels required mechanical clipping because electrocautery alone was insufficient.

The excised mass measured 35 × 13 × 4.5 cm. Two 10 French silicone drains were placed into the dead space, and a layered closure was achieved using absorbable sutures. Thereafter, incisional negative pressure wound therapy (PREVENA®; Solventum Intellectual Properties Company, Minnesota, USA) was applied to the surgical wound. Operative time amounted to 155 min with an estimated blood loss of 700 ml.

Postoperatively, oral tranexamic acid (1 g three times daily) was administered for 48 h. Haemoglobin dropped to 78 g/L on postoperative day 1. Due to anaemia-related symptoms during early postoperative mobilisation, transfusion of one unit of red blood cells was administered. Symptoms resolved after transfusion. Both drains and incisional negative wound dressing were removed on postoperative day 5, and the patient was discharged home the same day in stable condition. An abdominal compression garment was prescribed for six weeks postoperatively.

Recovery was uneventful, including normal wound healing and absence of seroma. Histopathology confirmed the benign origin of plexiform neurofibroma without atypia or malignancy. At 9 months following surgery, the patient reported well-being and satisfaction foremost regarding symmetry of the contour (Fig. 1b).

## Discussion

PNFs are challenging tumours because of their infiltrative growth pattern and dense vascularity. Surgical management is frequently limited to subtotal debulking aimed at improving function and/or appearance while preserving adjacent structures.^3^ Complete resection is often impossible, and recurrence remains common.^6^ Minimising intraoperative bleeding, therefore, remains a key surgical objective in optimising outcomes.

Bleeding represents one of the main operative risks during PNF surgery. Neurofibromin deficiency affects vascular endothelial and smooth muscle cells, resulting in the development of dysplastic vessels and eventually abnormal sinusoidal channels.^5^, ^6^, ^7^ These abnormalities may render electrocautery alone insufficient for haemostasis.

Several factors increase haemorrhagic risk during resection, including tumour size, anatomic location, previous surgery at the affected anatomical region and structural deformation.^4^ A recent retrospective study of 516 elective neurofibroma resections in NF1 patients demonstrated that a neurofibroma length of greater than 13 cm was associated with significantly higher rates of requiring blood cell transfusion.^8^ In the present case, the size and growing pattern of the tumour suggested substantial bleeding risk, emphasising the need for careful preoperative planning.

Mechanical haemostatic techniques played a central role intraoperatively. Although electrocautery allowed for tissue dissection, vascular clips were essential because large and infiltrated vessels within the adjacent soft tissues could not be neither safely isolated, nor coagulated effectively. Postoperative tranexamic acid was administered, reflecting our usual practice; however, there is no clear evidence supporting its benefit in this setting.^9^

Adjunctive techniques such as transcatheter arterial embolisation have been described to reduce surgery-related blood loss in selected cases, although their effectiveness may be limited by the diffuse and dysplastic vascular supply characteristic of PNFs.^5^ Other technologies, including vessel sealing systems, may also improve bleeding control.^4^

Despite recent advances in targeted therapies such as selumetinib for inoperable PNFs, surgery remains necessary for symptomatic or deforming lesions.^3^ In elderly patients, associated comorbidities may further complicate surgical management of these fragile tissues.

Malignant transformation of NF1 occurs in approximately 5–15% of all PNFs and should be suspected in cases of rapid tumour growth, pain, and/or neurological symptoms.^3^ MRI remains, therefore, essential not only for the exclusion of suspicious radiological features, but also for surgical planning.^6^

This case highlights the importance of anticipating major bleeding during PNF surgery and adapting haemostatic strategies intraoperatively. Given the paucity of literature on perioperative haemostatic strategies for large PNFs, this report aims to contribute to the discussion and raise awareness of these challenges for meticulous surgical planning and eventually patient safety. Principles and expertise derived from plastic and aesthetic surgery represent a valuable adjunct in complex tumour resections, not only by optimizing morphological outcomes and preserving body contour harmony, but also by contributing to improved quality of life and psychosocial well-being for patients following surgical management.

## Conclusion

Surgical treatment of PNFs in NF1 patients remains technically demanding because of their diffuse infiltrative growing pattern and abnormal vascularisation. This case illustrates the importance of individualised perioperative planning and meticulous haemostatic management. Anticipating haemorrhagic complications is therefore essential to minimise perioperative morbidity and optimise surgical outcomes.

## Ethical approval

Written informed consent was obtained from the patient for publication of this case report and accompanying images.

## Declaration of generative AI and AI-assisted technologies in the manuscript preparation process

During the preparation of this work the authors used [Nano Banana/Grammarly/ChatGPT] in order to assist with the creation of the anatomical illustration and to improve the linguistic and grammatical quality of the manuscript. After using these tools, the authors reviewed and edited the content as needed and take full responsibility for the content of the published article.

## Declaration of competing interest

This project did not receive any funding. None of the other authors have any disclosures relevant to the content of this work.
